# Case Report: Mutations in *JAK3* causing severe combined immunodeficiency complicated by disseminated Bacille Calmette–Guérin disease and *Pneumocystis* pneumonia

**DOI:** 10.3389/fimmu.2022.1055607

**Published:** 2022-11-17

**Authors:** Ying Pan, Hui Pan, Chunan Lian, Beiyan Wu, Jieying Lin, Guang Huang, Binglin Cui

**Affiliations:** ^1^ The Department of Pediatrics, The First Affiliated Hospital of Shantou University Medical College, Shantou, Guangdong, China; ^2^ The Outpatient Department, Shantou Longhu People’s Hospital, Shantou, Guangdong, China; ^3^ The Clinical Research Unit, Shantou University Medical College, Shantou, Guangdong, China

**Keywords:** *JAK3* deficiency, severe combined immunodeficiency, *Mycobacterium tuberculosis*, *Pneumocystis jirovecii*, case report

## Abstract

**Background:**

As a form of severe combined immunodeficiency (SCID), Janus kinase 3 (*JAK3*) deficiency can be fatal during severe infections in children, especially after inoculation of live-attenuated vaccines. We report a unique case of *JAK3* deficiency with two compound heterozygous *JAK3* mutations complicated by disseminated Bacille Calmette–Guérin (BCG) disease and *Pneumocystis* pneumonia.

**Case description:**

A 5-month-old Chinese girl presented with recurring fever and productive cough after BCG vaccination and ineffective antibiotic treatment. Chest CT demonstrated bilateral infiltrations, enlarged mediastinal and axillary lymph nodes, and hypoplasia of the thymus. *Mycobacterium tuberculosis* and *Pneumocystis jirovecii* were detected from blood samples by sequencing. Acid-fast bacilli were also found from the sputum aspirate and gastric aspirate. Lymphocyte subset analyses indicated T-B+NK- immunodeficiency, and gene sequencing identified two heterozygous missense mutations (one unreported globally) in the Janus homology 7 (JH7) domain of *JAK3*. The patient received rifampicin, isoniazid, ethambutol, and trimethoprim/sulfamethoxazole and was discharged after improvements but against advice.

**Outcome:**

The patient died at 13 months of age due to severe infections and hepatic damage.

**Discussion:**

SCID should be recognized before inoculation of live-attenuated vaccines in children. Newborn screening for SCID is advocated. Further investigations are needed to better understand the pathogenicity of the variants and molecular mechanism of the JH7 domain of *JAK3*.

## Introduction

Severe combined immunodeficiency (SCID) is a heterogeneous group of inherited disorder characterized by profound impairment in cellular and humoral immunity that can lead to death from severe infection(s) in infancy (1, 2). The incidence of SCID has been recorded as 1 in 58,000 live births based on a US newborn screening data (3, 4). According to immunologic phenotype classification, SCID can be grouped into four distinct categories: T-B+NK+ [T cell-deficient, normal B cells and natural killer (NK) cells], T-B+NK-, T-B-NK+, and T-B-NK- (1, 2). T-B+NK± SCIDs, accounting for 67%~74% of all SCIDs, are mostly triggered by pathogenic genetic variants affecting the common gamma chain (γ_c_) of interleukin 2 receptor (IL-2R), the associated downstream signaling enzyme Janus kinase 3 (*JAK3*), or IL-7R alpha chain gene (2, 5). As an unusual subtype comprising about 5% of all SCIDs (4, 6), *JAK3* deficiency with various *JAK3* mutations has been sporadically reported globally (6–8). Unlike X-linked γ_c_ SCID that generally affects male patients, *JAK3* deficiency is autosomal recessive inheritance and affects both male and female patients (2). The patients with SCID, including *JAK3* deficiency, have a noticeably high risk for severe, disseminated, and even fatal infections after inoculation of live-attenuated vaccines (5, 8–11), particularly Bacille Calmette–Guérin (BCG), being an active attenuated *Mycobacterium bovis* vaccine (5, 8, 10, 11). The incidence of disseminated BCG disease is approximately two cases per 1 million BCG-vaccinated children with a mortality rate of 80%, while the incidence of disseminated BCG disease is as high as one in every two BCG-vaccinated SCID patients (5, 11). *JAK3* deficiency complicated by BCG disease after BCG vaccination has been rarely reported worldwide (8, 12, 13). Herein, we describe a unique case of *JAK3* deficiency with two compound heterozygous *JAK3* mutations complicated by disseminated BCG disease and *Pneumocystis jirovecii* pneumonia (PJP).

## Case presentation

A 5-month-old female infant was admitted to the First Affiliated Hospital of Shantou University Medical College due to recurring fever and productive cough for 2 weeks unresponsive to cephalosporin and penicillin in a local hospital. She was born full-term with a weight of 2.7 kg (3 < P < 10). BCG vaccine, routinely administered at birth in China, was given at 2 months of age because of meconium aspiration pneumonia after birth. She had been hospitalized locally for 7 days for generalized small papules and pustules at 4 months and recovered gradually after supportive treatment. No tuberculosis contact was noted. Her non-consanguineous parents and only older brother were all healthy. There was no family history of similar or genetic diseases. Her total white blood cell count, neutrophil count percentage, and C-reactive protein were apparently elevated along with a low to normal lymphocyte count; chest radiograph showed bilateral infiltrations (thymus shadow not mentioned) before her current admission.

On physical examination, her weight was 5.2 kg (<P1). Several enlarged axillary lymph nodes were palpable bilaterally. Her oral mucosa was intact. Coarse breath sounds and rhonchi were audible bilaterally. Throat swab was positive for parainfluenza virus (PIV) and respiratory syncytial virus (RSV) by PCR assays, while routine cerebrospinal fluid analysis/culture and blood HIV testing were all negative. Chest CT revealed bilateral parenchymal and interstitial infiltrations, multiple enlarged lymph nodes in the mediastinum and right axilla, and hypoplasia of the thymus.

The patient was treated sequentially with intravenous ceftriaxone, piperacillin/tazobactam, and vancomycin, but her symptoms were not relieved.

**Figure 1:**
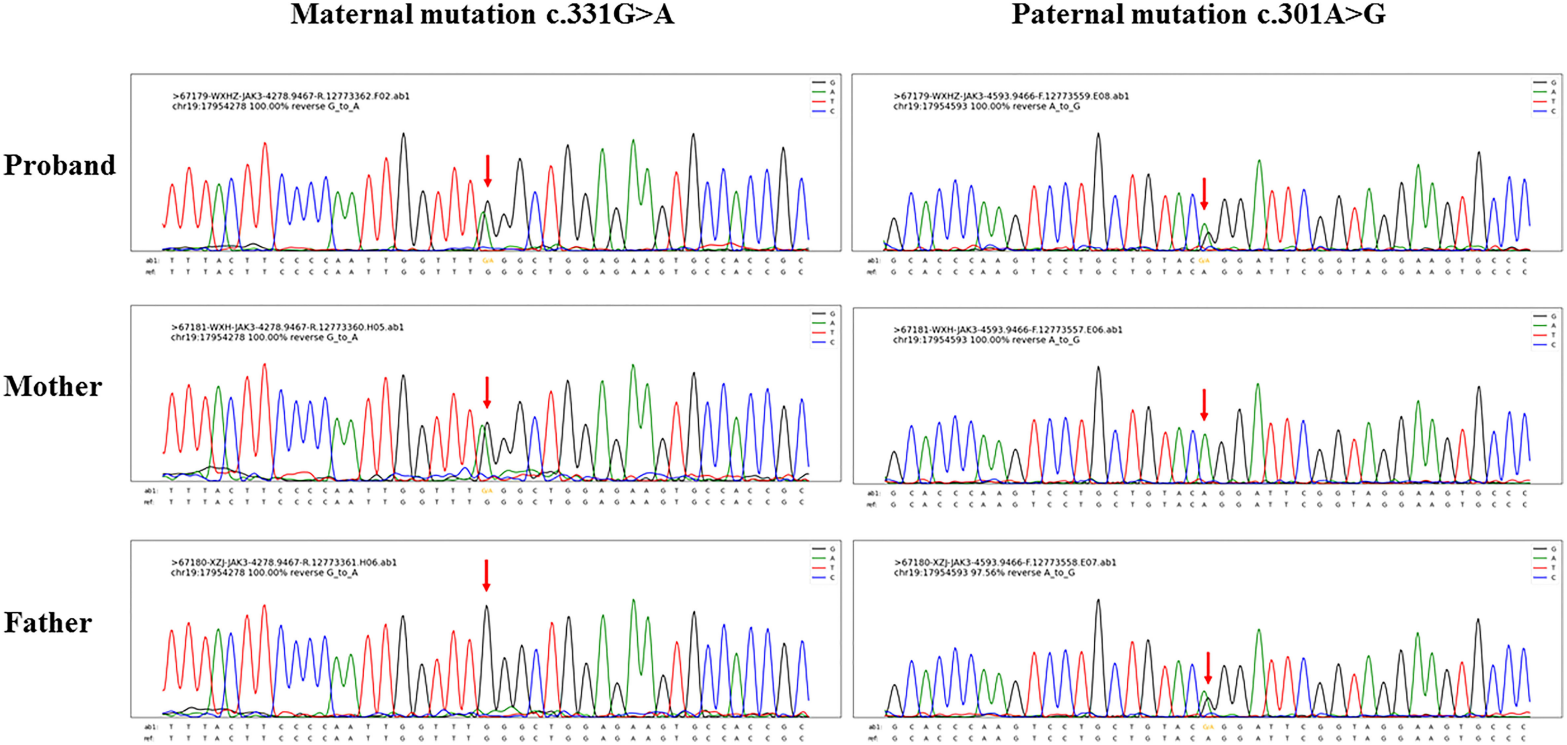
Gene sequencing results of the index patient of Janus kinase 3 (*JAK3*) deficiency and her parents. Sequencing detected two missense mutations both located in the Janus homology 7 (JH7) domain of the *JAK3* gene, as maternal mutation c.331G>A and paternal mutation c.301A>G.

From her blood samples, the sequences of *M. tuberculosis* and *P. jirovecii* were mapped through next-generation sequencing. Acid-fast bacilli were found in the concentrated smears of the sputum aspirate and gastric aspirate; *M. tuberculosis* and *P. jirovecii* were not isolated after culturing. The subtypes of *M. tuberculosis* failed to be identified prior to antituberculosis therapy. To clarify the source of tuberculosis infection, her older brother, parents, and grandparents underwent chest X-ray examinations and purified protein derivative (PPD) skin tests, which were all negative. In addition, low numbers of T cells and natural killer (NK) cells and increased B lymphocytes from lymphocyte subset analyses confirmed T-B+NK- immunodeficiency (**Table 1**). Subsequent gene sequencing identified two heterozygous missense mutations in the Janus homology 7 (JH7) domain of the *JAK3* gene of the patient, each inherited from her parents (**Figure 1**). The paternal mutation C.301A>G at nucleotide 301 encoded by exon 3 changed amino acid 101 from arginine to glycine (i.e., p.Arg101Gly), and the maternal mutation C.331G>A at nucleotide 331 encoded by exon 4 altered amino acid 111 from glycine to arginine (i.e., p.Gly111Arg). These two mutations were predicted to be pathogenic by Sorting intolerant from tolerant (SIFT) (both <0.05, deleterious), PolyPhen2_HVAR (paternal: 0.997, maternal: 0.992; both probably damaging), PolyPhen2_HDIV (both >0.957, probably damaging), and Rare exome variant ensemble learner (REVEL) (paternal: 0.809, maternal: 0.655; both pathogenic).

**Table 1:** Patient laboratory profiles after admission.

Variable	Day 1	Day 5	Day 11	Day 18	Day 25	Reference range
**Complete blood count**						
RBCs (10^12^ cells/L)	4.78	4.58	4.29	5.01	4.39	4.00-5.50
Hemoglobin (g/L)	116	113	103	121	109	97-141
WBCs (10^9^ cells/L)	***23.99***	***20.14***	***23.92***	***20.74***	8.54	4.80-14.60
Neutrophils (10^9^ cells/L)	***19.17***	***16.35***	***18.47***	***12.71***	***6.43***	0.80-6.40
Lymphocytes (10^9^ cells/L)	2.67	***1.87***	2.92	5.25	***0.80***	2.50-9.00
Monocytes (10^9^ cells/L)	***1.96***	***1.79***	***2.06***	***2.38***	0.99	0.17-1.06
Platelets (10^9^ cells/L)	534	420	***613***	503	293	190-579
**CRP (mg/L)**	***80.78***	***53.86***	***16.90***	1.39	***13.60***	<10.00
**PCT (ng/ml)**	***0.28***	–	–	–	–	0-0.05
**Liver function**						
Lactate dehydrogenase (U/L)	***545***	***457***	***491***	***335***	***260***	120-250
Aspartate transaminase (U/L)	45.33	44.21	***106.73***	***94.52***	39.39	21.00-80.00
Alanine aminotransferase (U/L)	18.79	14.98	26.49	64.39	10.47	8.00-71.00
Gamma-glutamyl transpeptidase (U/L)	***46.09***	***43.35***	***56.56***	***500.64***	***210.58***	6.00-31.00
Total protein (g/L)	62.51	60.35	59.09	***54.52***	***53.23***	55.00-75.00
Albumin (g/L)	***33.23***	***32.01***	***33.45***	***30.88***	***32.81***	39.00-54.00
Globulin (g/L)^#^	29.28	28.34	25.64	23.64	20.42	10.00-30.00
Total bilirubin (μmol/L)	3.44	4.33	5.23	***25.28***	9.62	1.70-20.50
Direct bilirubin (μmol/L)	0.92	0.97	1.63	***14.99***	4.32	0-6.00
Indirect bilirubin (μmol/L)	2.52	3.36	3.60	10.29	5.30	0-14.00
***Immunoglobulin***						
IgG (g/L)	***4.06***	–	–	–	–	7.51-15.60
IgM (g/L)	0.53	–	–	–	–	0.46-3.04
IgA (g/L)	***<0.07***	–	–	–	–	0.82-4.53
***Lymphocyte subset***						
CD3+ (%)	–	***0.69***	–	***3.56***	–	50.00-84.00
CD45+CD3+CD4+ (%)	–	***0.25***	–	***3.00***	–	27.00-51.00
CD45+CD3+CD8+ (%)	–	***0.01***	–	***0.54***	–	15.00-44.00
CD4+/CD8+ (%)	–	***25.00***	–	***5.56***	–	0.98-1.94
CD45+CD3-CD19+ (%)	–	***94.04***	–	***94.14***	–	5.00-20.00
CD45+CD3-CD16+CD56+ (%)	–	***2.76***	–	***1.15***	–	8.00-28.00
CD3+ (cells/μL)	–	***13***	–	***187***	–	955-2,860
CD45+CD3+CD4+ (cells/μL)	–	***5***	–	***158***	–	550-1,440
CD45+CD3+CD8+ (cells/μL)	–	***<1***	–	***28***	–	320-1,250
CD45+CD3-CD19+ (cells/μL)	–	***1,759***	–	***4,942***	–	90-580
CD45+CD3-CD16+CD56+ (cells/μL)	–	***52***	–	***60***	–	150-900

^#^Patient received intravenous immunoglobulin prior to admission. Abnormal values are italicized and bolded.

*JAK3*, Janus kinase 3; RBC, red blood cell; WBC, white blood cell; CRP, C-reactive protein; PCT, procalcitonin; CD3+, T cells; CD45+CD3+CD4+, helper-inducer T cells; CD45+CD3+CD8+, suppressor-killer T cells; CD45+CD3-CD19+, B cells; CD45+CD3-CD16+CD56+, NK, natural killer cells.

Consequently, her treatment strategy was switched to rifampicin, isoniazid, and ethambutol for tuberculosis, intravenous immunoglobulin to enhance immune functions, and further trimethoprim/sulfamethoxazole for PJP, after which improvements in symptoms (cough and fever) and lab results (e.g., decreased serum lactate dehydrogenase) were noted. After being hospitalized for 28 days, the patient improved but was discharged upon request of her parents who were satisfied with the timely diagnosis and treatment. During our follow-up, the patient continued antituberculosis treatment routinely at the assigned local tuberculosis prevention clinic and died without hematopoietic stem cell transplantation (HSCT) or autopsy at 13 months of age mainly because of severe infections in addition to hepatic injury (autopsy is culturally unacceptable and therefore uncommon in China). Her other laboratory results are shown in **Table 1**. Written informed consent for laboratory examinations and submission/publications of this work was obtained from her parents. This study was approved by the Ethics Committee of the First Affiliated Hospital of Shantou University Medical College.

## Discussion

**Figure 2:**
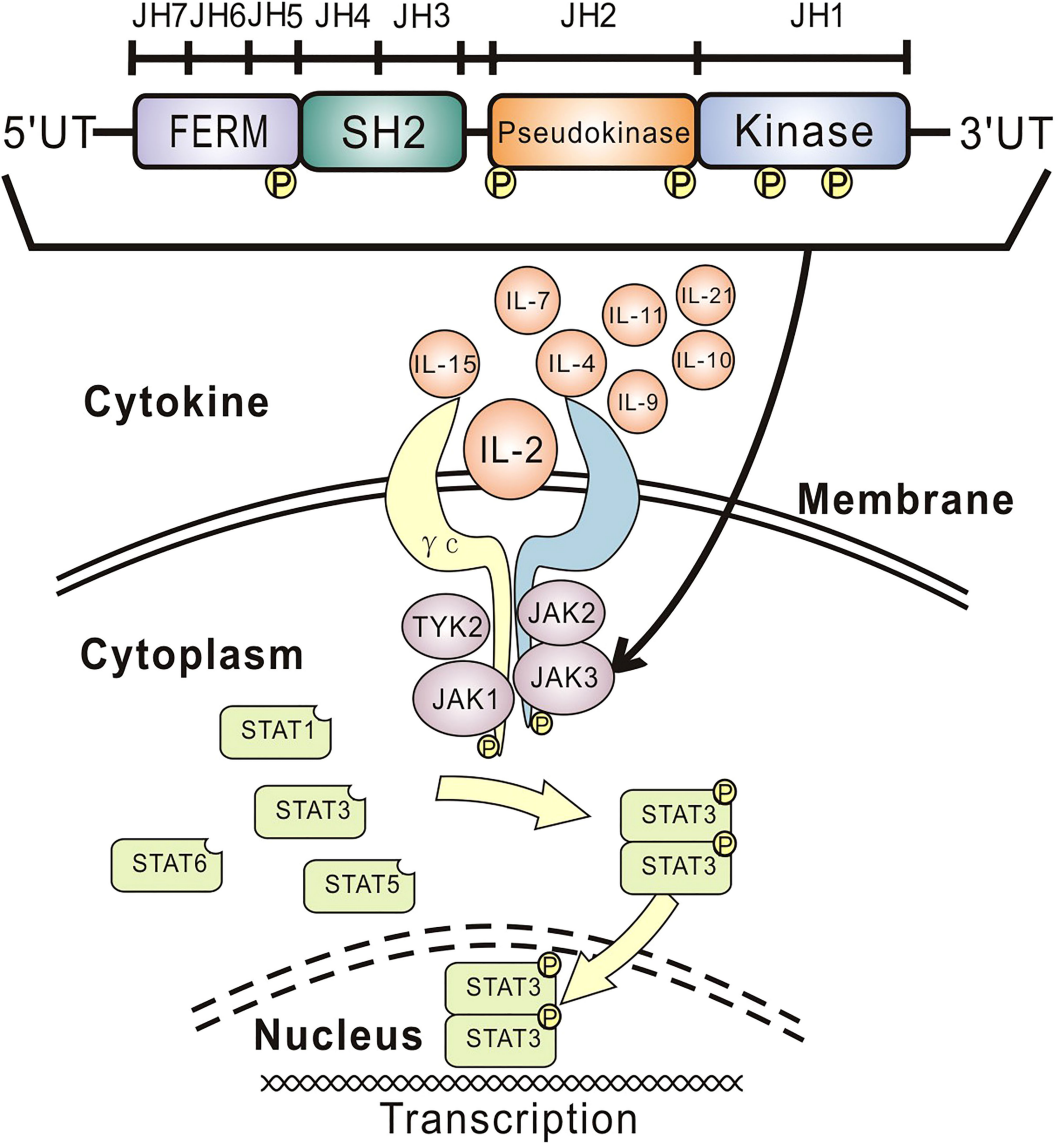
Overview of cytokine signaling and *JAK3* structure. Intracellular activation signal through cytokine-γc-*JAK3* binding induces *JAK3* to phosphorylate STAT proteins, which therefore dimerize and translocate to the nucleus to initiate the transcription program. The JAK family includes four homologs (i.e., JAK1, JAK2, *JAK3*, and TYK2), each containing seven conserved JH domains. The JH1 (kinase) domain regulates kinase activity, and the JH2 (pseudokinase) domain is essential for normal *JAK3* functions, including catalytic activity and autophosphorylation. The SH2-like JH3, JH4, and JH5 domains are considered imperative for the *in vivo* assembly of the JAKs. The JH5, JH6, and JH7 domains (namely, FERM) are required for binding to γc for signal transduction and regulating catalytic activity. JAK, Janus kinase; γc, gamma chain; STAT, signal transducer and activator of transcription; JH, Janus homology; IL, interleukin; FERM, four-point-one, ezrin, radixin, moesin; SH2, Src homology-2; TYK2, tyrosine kinase 2.

The human *JAK3* gene is predominantly expressed in lymphoid and myeloid cells and located in chromosome 19p12-13.1, consisting of 23 exons and an open reading frame of 3,372 nucleotides (1, 2, 14–17). Tyrosine kinase *JAK3* binds specially to the γ_c_ subunit, an essential component of the cytokine (i.e., IL-2, IL-4, IL-7, IL-9, IL-10, IL-11, IL-15, and IL-21) receptor complexes (**Figure 2**) (1, 7, 15, 18). Intracellular activation signal through cytokine-γ_c_-*JAK3* binding induces *JAK3* to phosphorylate signal transducer and activator of transcription (STAT) proteins (i.e., STAT1, STAT3, STAT5, and STAT6) (18, 19), which therefore dimerize and translocate to the nucleus to initiate the transcription program (1, 7). While IL-2, IL-4, and IL-9 play crucial roles in the development of B cells and T cells (1, 14, 20), IL-7 and IL-15 mainly regulate the differentiation and activation of T cells and NK cells (1, 2, 6, 14, 21). In consequence, *JAK3* deficiency can lead to serious defects of T cells and NK cells with a normal or an increased number of poorly effective B cells (i.e., T-B+NK-) and a clinical phenotype nearly identical to that of γ_c_ deficiency (2, 6, 16). In our case, the profiles of lymphocyte subsets and the obviously decreased levels of immunoglobulins A and G are concordant with the manifestations of *JAK3* deficiency.

The JAK family consists of four mammalian homologs (i.e., JAK1, JAK2, *JAK3*, and TYK2) (17, 22), each containing 1,100–1,200 amino acids arranged in seven conserved JH domains according to sequence similarity (**Figure 2**) (15, 17). The C-terminal JH1 (kinase) domain plays a key role in regulating kinase activity (7). The JH2 (pseudokinase) domain, despite its lack of kinase activity, binds to the JH1 domain and thereby is essential for normal *JAK3* functions, including catalytic activity and autophosphorylation (6, 7, 23). The majority of reported pathogenic mutations leading to SCID, including *JAK3* deficiency, occur in the JH1 and JH2 domains, since they constitute nearly half of the whole JAK sequence (6, 7, 14, 24). The JH3, JH4, and JH5 domains are considered imperative for the *in vivo* assembly of the JAKs (6, 14). The N-terminal JH5, JH6, and JH7 domains (namely, four-point-one, ezrin, radixin, moesin (FERM)) are required for binding to γ_c_ for signal transduction and regulating catalytic activity (6, 14), of which JH7 and the head of JH6 (i.e., amino acids 1–193) of *JAK3* are sufficient for binding to the BOX1 (a highly conserved motif) proline residues 266 and 269 of γ_c_ specifically in response to IL-2 (22, 25). In our case, the maternal mutation C.331G>A has not been reported globally, while the paternal mutation C.301A>G has been listed at low frequency (i.e., 0.00006157) in the Genome Aggregation Database (gnomAD; http://www.gnomad-sg.org/). These mutations were predicted to be pathogenic by different predicting tools. Single/multiple amino acid substitutions in the JH7 domain naturally occurring in *JAK3* deficiency patients or from *JAK3*-JAK1 or *JAK3*-JAK2 chimeras can prevent kinase–receptor interaction and IL-2-induced signaling (6, 7, 22, 24, 25). Similarly, the two missense mutations in the JH7 domain of our patient may affect IL-2-γ_c_-*JAK3* binding and thus the DNA transcriptions of B cells and T cells. Point mutations only in the JH7 domain of *JAK3* observed in our patient are speculated to affect the binding of *JAK3* to γ_c_ and other upstream cytokines, probably IL-7 and/or IL-15, therefore contributing to the reduced number of NK cells. Considering HSCT, the best remedy for *JAK3* deficiency, is still not completely successful for restoring the functions of B cells and NK cells (6); our findings call for further investigations to better understand the role/mechanism of the JH7 domain of the *JAK3* gene in regulating the functions of NK cells, which would be helpful for improving HSCT and gene therapy in the future.

The manifestations and clinical outcomes of *JAK3* deficiency due to different mutations in the JH1–7 domains are nearly identical (6, 8, 26). Similar to other SCIDs, *JAK3* deficiency patients normally present with recurrent severe respiratory infections, refractory diarrhea, thrush, and/or retarded growth, characterized by irrecoverable infections mostly caused by opportunistic and/or multiple pathogens (2, 5, 6, 8, 14) or live-attenuated vaccination (5, 6, 8–11). Although we were unable to further identify the *M. tuberculosis* subspecies, the bloodstream/multisite tuberculosis infection following BCG vaccination and a lack of tuberculosis contact in the infant support a diagnosis of disseminated BCG disease, consistent with the definition of disseminated BCG disease (10–12). Along with other reports (8, 12, 13), our case highlights the severe consequences of SCID and disseminated BCG disease.

The inoculation of BCG vaccine has been implemented as an essential and generally safe means to prevent tuberculosis infection, mostly given at birth in developing countries, including China (http://www.bcgatlas.org/). However, primary immunodeficiency diseases (PIDs) should be ruled out before BCG vaccination, as disseminated BCG disease is usually severe and life-threatening for PID patients (5). *M. bovis* is the cause of BCG disease, naturally resistant to pyrazinamide (11). Hence, anti-*M. bovis* therapy basically comprises isoniazid, rifampicin, ethambutol, and streptomycin (5, 11, 12). The compromised liver function of our patient led to a final choice of triad therapy being isoniazid, rifampicin, and ethambutol based on the literature (5, 11, 12). Indeed, early screening for PIDs in children will be definitely better to avoid inoculation of live-attenuated vaccines and consequently reduce vaccine-related mortality (5). Neonatal PID screening programs, such as T cell receptor excision circles (TREC)-based newborn screening (2), have been deployed in some developed countries/regions (8) but unfortunately not in mainland China. However, absolute lymphopenia (i.e., absolute lymphocyte count <3,000/mm^3^) in a newborn complete blood count should alert us to screen for and exclude SCID (27).

The fungus *P. jirovecii*, commonly existing in ambient environments, can cause PJP as a serious and fatal opportunistic infection more commonly in HIV patients than in SCID patients (26). The symptoms of PJP usually last for several days/weeks, including fever, cough, difficulty breathing, chest pain, chills, and fatigue. Elevated serum lactate dehydrogenase is one of the most remarkable laboratory findings for PJP and other fungal infections (28). Chest radiograph can show diffuse bilateral interstitial infiltrates, while chest CT may reveal ground-glass attenuation or cystic lesions (28). As *Pneumocystis* is extremely difficult to culture, conclusive diagnosis basically needs detection/identification of the organism by PCR, dye staining, or fluorescein antibody staining of respiratory specimens (28). Trimethoprim/sulfamethoxazole is the drug of choice for preventing/treating PJP in immunocompromised patients. In our case, *Pneumocystis* as an air-borne transmitted pathogen, recurring fever and cough, *P. jirovecii* detected in blood, serum lactate dehydrogenase changes during the course, bilateral parenchymal and interstitial infiltrations shown by chest CT (despite the influence of simultaneous infection of *M. tuberculosis*), and effective response to trimethoprim/sulfamethoxazole treatment support a diagnosis of PJP. This is the second global report of *JAK3* deficiency and PJP, aside from a Japanese case (26). PIV and RSV detected from the throat specimen by PCR alone in our patient could not be confirmed as current or past infections or just colonization.

Mainstream therapy regimen for SCID includes antimicrobial drugs, immunoglobulin replacement, enzyme replacement, retroviral gene therapy, and finally HSCT as a life-saving but not perfect modality (6, 14, 24). The outcome of gene therapy for *JAK3* deficiency is still unsatisfactory (6). Nearly all SCID patients die before 2 years of age, unless effective therapies are utilized to reconstruct their immune systems (2). Our patient died without HSCT at 13 months of age as a consequence of severe infections and hepatic damage.

There are several limitations in this study. *M. bovis* as the cause of the disseminated BCG disease failed to be identified in advance of antituberculosis treatment. After ruling out the possibilities of tuberculosis contact/infection in all of her family members, *M. bovis* was highly likely to be the only cause of the infection. Although the observed mutations were predicted to be pathogenic, further basic research (6, 7, 25, 29) is still needed to examine the actual pathogenicity of the variants.

## Conclusions

This report has elucidated an exceptional case of *JAK3* deficiency with two *JAK3* mutations complicated by disseminated BCG disease, *Pneumocystis* pneumonia, and probable respiratory viral infections. If possible, SCID should be identified before inoculation of live-attenuated vaccines in children. Screening for SCID in neonatal lymphopenia is suggested for developing countries such as China. Further basic/clinical studies are required to understand the pathogenicity of the variants and molecular mechanism of the JH7 domain of the *JAK3* protein for better gene therapy and HSCT.

## Data availability statement

The raw sequence data (GSA-Human: HRA002763) for this study have been deposited in the Genome Sequence Archive in National Genomics Data Center, China National Center for Bioinformation/Beijing Institute of Genomics, and Chinese Academy of Sciences (https://ngdc.cncb.ac.cn/gsa-human) 30, 31).

## Ethics statement

The studies involving human participants were reviewed and approved by Ethics Committee of the First Affiliated Hospital of Shantou University Medical College. Written informed consent to participate in this study was provided by the participants’ legal guardian/next of kin.

## Author contributions

YP, HP, and BC wrote the manuscript, performed analyses, and revised the manuscript. YP, CL, BW, JL, GH, and BC provided clinical information. CL, BW, JL, and GH provided critical discussion. BC conceptualized the study and revised the manuscript. All authors contributed to the work and approved the final version of the manuscript.

## Funding

This study was funded by the 2020 Guangdong Provincial Funds (grant no. 2020A111129021).

## Acknowledgments

We are grateful to Dr. Frieda Law (Li Ka-Shing Foundation, Consultant of Shantou University Medical College, Shantou, Guangdong, 515041, P.R. China) for invaluable suggestions on revising the manuscript.

## Conflict of interest

The authors declare that the research was conducted in the absence of any commercial or financial relationships that could be construed as a potential conflict of interest.

## Publisher’s note

All claims expressed in this article are solely those of the authors and do not necessarily represent those of their affiliated organizations, or those of the publisher, the editors and the reviewers. Any product that may be evaluated in this article, or claim that may be made by its manufacturer, is not guaranteed or endorsed by the publisher.
